# Diversity of heterotrimeric G-protein γ subunits in plants

**DOI:** 10.1186/1756-0500-5-608

**Published:** 2012-10-31

**Authors:** Yuri Trusov, David Chakravorty, José Ramón Botella

**Affiliations:** 1 Plant Genetic Engineering Laboratory, School of Agriculture and Food Sciences, University of Queensland, Brisbane, Queensland, 4072, Australia; 2 Biology Department, 208 Mueller Laboratory, Pennsylvania State University, University Park, Pennsylvania, 16802, USA

**Keywords:** Heterotrimeric G-proteins, Signal transduction, Prenylation, S-acylation

## Abstract

**Background:**

Heterotrimeric G-proteins, consisting of three subunits Gα, Gβ and Gγ are present in most eukaryotes and mediate signaling in numerous biological processes. In plants, Gγ subunits were shown to provide functional selectivity to G-proteins. Three unconventional Gγ subunits were recently reported in Arabidopsis, rice and soybean but no structural analysis has been reported so far. Their relationship with conventional Gγ subunits and taxonomical distribution has not been yet demonstrated.

**Results:**

After an extensive similarity search through plant genomes, transcriptomes and proteomes we assembled over 200 non-redundant proteins related to the known Gγ subunits. Structural analysis of these sequences revealed that most of them lack the obligatory C-terminal prenylation motif (CaaX). According to their C-terminal structures we classified the plant Gγ subunits into three distinct types. Type A consists of Gγ subunits with a putative prenylation motif. Type B subunits lack a prenylation motif and do not have any cysteine residues in the C-terminal region, while type C subunits contain an extended C-terminal domain highly enriched with cysteines. Comparative analysis of C-terminal domains of the proteins, intron-exon arrangement of the corresponding genes and phylogenetic studies suggested a common origin of all plant Gγ subunits.

**Conclusion:**

Phylogenetic analyses suggest that types C and B most probably originated independently from type A ancestors. We speculate on a potential mechanism used by those Gγ subunits lacking isoprenylation motifs to anchor the Gβγ dimer to the plasma membrane and propose a new flexible nomenclature for plant Gγ subunits. Finally, in the light of our new classification, we give a word of caution about the interpretation of Gγ research in Arabidopsis and its generalization to other plant species.

## Background

Heterotrimeric G-proteins, consisting of three distinct subunits: Gα, Gβ and Gγ, constitute one of the most important signal transduction systems in eukaryotes. According to the classic paradigm, ligand-bound G-protein coupled receptors catalyze the exchange of GDP for GTP on the Gα subunit, resulting in activation of the heterotrimer and dissociation of the two functional elements, the Gα subunit and the Gβγ dimer. Gα and Gβγ independently interact with multiple downstream effectors mediating specific signal transduction pathways until the intrinsic GTPase activity of the Gα subunit hydrolyzes GTP to GDP, which increases Gα affinity for the Gβγ dimer, resulting in re-association of the heterotrimer at the receptor [1-3].

Plant heterotrimeric G-proteins are involved in multiple physiological processes [4-8]; however the available set of subunits is limited. In the fully sequenced Arabidopsis genome only one gene is present for the Gα and Gβ subunits [5,9], while three genes are now known for Gγ subunits [10-13]. With single Gα and Gβ subunits, it is logical to assume that Gγ is solely responsible for providing functional selectivity to the heterotrimer, as has been proven for Arabidopsis [10,14,15]. In some plants two to four canonical Gα and Gβ subunits have been identified [16-18], however their functional specificity is yet to be studied. At the same time, in one of these species, soybean, the set of Gγ subunits has been expanded to 10 [19].

The Gγ subunit is an essential part of the heterotrimer, binding tightly to Gβ and anchoring the Gβγ dimer to the plasma membrane [1,20-23]. Most of the known Gγ subunits are relatively small proteins of about 8–11 kDa [11,12,24-26]. They contain a conserved prenylation signal at their C-termini, which is a target for post-translational prenylation [1,3,22,27,28]. This modification considered to be crucial for anchoring the Gβγ dimer to the plasma membrane and for the entire heterotrimer function [29-32]. Recently, a novel Gγ subunit has been described in Arabidopsis, AGG3, containing a large cysteine-rich C-terminus, increasing the size of the protein to approximately 25 kDa (251 amino acids) [10]. On the other hand, one of the reported rice Gγ subunits, RGG2, does not contain a C-terminal prenylation signal [26] and similar variants were recently reported in soybean [19]. To the best of our knowledge, such severe deviations from the canonical structure have not been reported for any animal or fungal Gγ subunits.

It was found that the protein structure of the Gγ variants could differ dramatically within a single species [10,19,26], however the extent of these proteins’ diversity in plants and their phylogenetic relationships have not yet been studied. In this study we have identified over 200 non-redundant sequences of Gγ subunits from seed bearing plants and analyzed their structure and phylogenetic relationships. Based on their C-terminal amino acid sequences three distinct types were revealed. A robust phylogenetic analysis suggested a common origin for the three structural types within the plant kingdom. A new flexible nomenclature for plant Gγ subunits is suggested.

## Results

### Identification of plant Gγ subunits

Gγ subunits have been characterized only in four flowering plant species: Arabidopsis [10-12], rice [26], garden pea [18] and soybean [19]. In order to identify additional Gγ sequences we queried the “nucleotide collection (nr/nt)”, “expressed sequence tags (est)”, “high throughput genomic sequences (HTGS)” and “whole-genome shotgun reads (wgs)” databases in GeneBank using the BLAST (tblastn) algorithm [33] with the Arabidopsis and rice Gγ subunit protein sequences as queries. The published PsGγ1 and PsGγ2 sequences from garden pea show very low overall similarity to Gγ subunits from other species and lack the most of highly conserved residues including the DPLL motif present in all established plant and animal Gγ subunits. At the same time we found one pea EST showing high similarity to Gγ-encoding ESTs from other plants and particularly to other leguminous species, suggesting that the published PsGγ1 and PsGγ2 have a different origin and therefore we did not include them in this study. In rice, in addition to the two reported Gγs, RGG1 and RGG2, we have identified three other proteins sharing high similarity with the Arabidopsis Gγ subunit AGG3. Two of them, *GRAIN SIZE 3* (*GS3*) and *DENSE AND ERECT PANICLE1* (*DEP1*), were previously characterized but not identified as Gγ subunits [34-36]. These proteins were recently reclassified as Gγ subunits due to their high similarity with the experimentally proven Arabidopsis AGG3 subunit [10,37].

To exhaust the screening, newly found sequences from different plant taxa were translated into protein sequences and used as queries in additional BLAST searches. In total, over 300 non-redundant sequences representing plant Gγ subunits from land plants were obtained with more than one subunit found in most species. After discarding short and incomplete sequences, around 200 full-length or near full-length sequences were selected for analysis.

### Structural analysis reveals three types of plant Gγ proteins

**Figure 1:**
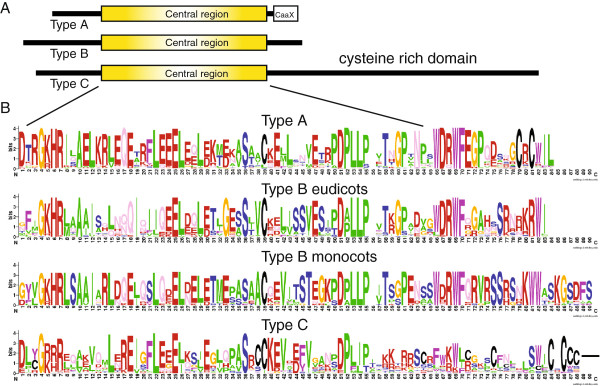
**General structure and conserved domains of plant Gγ subunits.** (**A**) Schematic representation of the three types of plant Gγ subunits. (**B**) WebLogo of the conserved central and C-terminal domains of Gγ subunits. Eudicot and monocot type B sequences were separated due to length and sequence difference of the C-terminal regions.

Initial inspection of aligned putative Gγ proteins revealed that the central part of the molecule was the most conserved, while N-terminal and C-terminal regions were variable. High variability in the N-termini has also been reported for animal Gγ subunits [38], but C-terminal variability seems to be a unique characteristic of plant Gγ subunits. Importantly, the variability observed in the C-termini was not random and three distinct types could be clearly identified. Based on their C-terminal amino acids, plant Gγ subunits can be divided into three structural types A, B and C. A schematic representation of the types is shown on Figure 1A. The central domain retains high sequence similarity in all three types indicating a close relationship and suggesting a conserved function for the domain. Multiple sequence alignments of the central domain and C-terminal region of types A and B are shown in Figure 1B, while only the central domain and a short fragment of the C-terminus of type C is shown in the Figure. The N-termini of the proteins were too variable to be unambiguously aligned, while the C-terminal region of type C subunits was too long to be presented in the Figure.

Type A Gγ subunits are small proteins (approximately 100 amino acids) containing the conserved C-terminal CaaX motif, known as a prenylation motif, which is a characteristic of all non-plant and conventional plant Gγ subunits. This type is similar to all other eukaryotic Gγ subunits reported so far and therefore could be considered as the archetypal heterotrimeric Gγ subunits. Considering the presence of the putative prenylation motif, we have defined type A Gγ subunits as short proteins with a potential site for post-translational prenylation. Representatives of type A were found in gymnosperms and flowering plants. Notably, in rosid species there are at least two variants of this type related to the Arabidopsis AGG1 and AGG2 proteins. At the same time most asterid species have only one Gγ subunit of this type.

The second Gγ subunit type, type B, has a substantial level of amino acid sequence similarity to the type A Gγ subunits, however, lacks the C-terminal CaaX box. Moreover, there is not a single cysteine residue in the proximity of the C-terminus. This type was found only in flowering plants, but not in gymnosperms. Surprisingly, no representatives of this type were identified in the fully sequenced Arabidopsis genome or in other intensively studied *Brassicaceae* genera, such as *Brassica* and *Raphanus*. Nevertheless, this gene was found in *Carica papaya* which belongs to the same order *Brassicales*, but to a different family *Caricaceae*, suggesting that the gene was probably lost in the *Brassicaceae* ancestor. The consensus C-terminus among eudicot sequences of this type is SRxxKRWI, while in monocot species the C-terminal consensus is KGSDFS (Figure 1B). Since none of these proteins contains cysteines in the C-terminal region, we have designated this type as non-prenylated Gγ subunits.

The third type, type C, accommodates Gγ subunits with relatively large (~70-350 amino acids) C-terminal extensions. Despite the pronounced variability in the C-terminus, sequences of this type share a considerable level of similarity with type A and B Gγ subunits in the central region (Figure 1B). Importantly, they contain all, but one, of the conserved residues forming hydrogen bonds and hydrophobic contacts in the interaction between Gγ and Gβ (amino acids L/V12, E25, S36, D/E51, P52, L53, L/I54 in Figure 1B). These residues are also conserved in mammalian Gγs [39]. Physical interaction with the Gβ subunit, plasma membrane localization and genetic analysis in Arabidopsis confirmed the type C representative, AGG3, as a *bona fide* Gγ subunit [10,13]. The C-terminal regions of type C Gγ subunits have an extremely high content of cysteine residues (19 to 38%) and their lengths are quite variable even between closely related species (Additional file 1: Table S1). The cysteine residues are distributed randomly along the region without common recognizable domains. Repeats of 5–10 amino acids are relatively common for the cysteine rich region. For instance in cotton (*Gossypium raimondii*) twelve tandem repeats with sound identity can be found, suggesting recent amplification events (Additional file 1: Figure S1). Alignment of Arabidopsis and *Brassica rapa* sequences revealed at least 6 indels ranging from 3 to 51 bp (Additional file 1: Figure S2). Conifers and cycads contain the shortest cysteine-rich regions, being of similar size even among species from different families (Additional file 1: Table S1).

### Phylogenetic relationships within plant Gγ subunits

**Figure 2:**
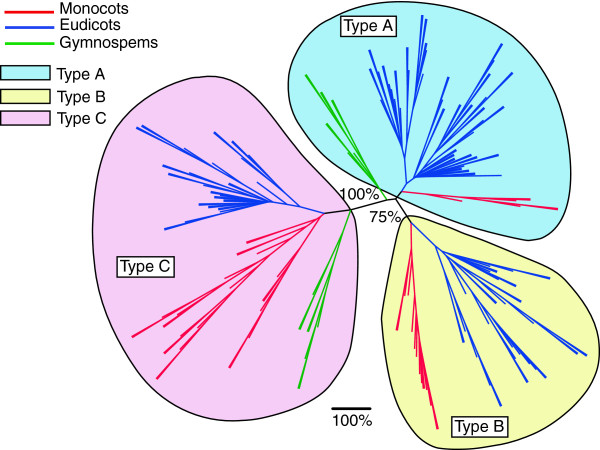
**Phylogenetic relationships of the three types of plant Gγ subunits.** Consensus tree representing 1000 unrooted bootstrap trees generated by protein parsimony algorithm; percentage shows statistically significant support for clusters corresponding to the structural types.

To investigate the generic relationships between the four identified types of plant Gγ subunits we performed phylogenetic analysis. As mentioned before, the central part of the protein was the most conserved in size and amino acid sequence. The N-termini varied in size from roughly 10 to 75 amino acids, while the C-termini varied even more dramatically, from 20 to 350 amino acids, effectively preventing any reasonable alignment within these two regions. Therefore only the conserved central domain was used for phylogenetic analysis. The protein sequences were aligned using the CLUSTAL-OMEGA software [40] and adjusted manually. We built and compared unrooted trees using different algorithms provided in the PHYLIP 3.66 package [41] as well as maximum likelihood algorithm using the PhyLM 3.0 package [42,43]. The obtained trees exhibited some variation in topology and low overall statistical support (bootstrap values for PHYLIP algorithms and approximate likelihood ratio test for branches (aLRT) for PhyLM)). This was not an unexpected outcome since we used fairly short sequences. Nonetheless, most of the trees showed significant statistical support for major clusters corresponding to the proposed structural types. Therefore, although limited, very important phylogenetic information could be retrieved. Parsimony and maximum likelihood trees displayed very similar topology and provided the best fit for the currently established plant phylogeny within the sub-trees of type A, B and C [44]. For demonstration purposes we selected the parsimony tree (Figure 2) due to its higher statistical support for the designated types. The parsimonious algorithm separated type C sequences in 100% of bootstrap trees. Type B sequences were sub-clustered within type A sequences with significant (75%) bootstrap support (Figure 2).

The phylogenetic analysis, thus, suggests that type B derived from type A sometime after flowering plants diverged from gymnosperms, but prior to the origin of monocots. This scenario corresponds well with our data mining results showing presence of type B only in flowering plants. Types A and C seem to originate before seed bearing plants diverged; in accordance, these types are present in gymnosperms and flowering plants. Several relatively recent duplication events took place within the types A and C resulting in the modern day diversity observed in plant Gγ subunits.

### Gγ gene structure comparison between plants and other eukaryotes

Plant and animal Gγ subunits share limited sequence similarity even at the protein level [10,11,39]. In order to obtain a different insight on the relationship among plant Gγ subunit genes and to establish a degree of relatedness between plant and other eukaryotic Gγ genes, we compared intron-exon structures of Gγ genes from several species representing different eukaryotic domains (Additional file 1: Figure S3). The intron-exon structure further supported the closer relationship between all plant Gγ subunits. All tested type A and B Gγ genes have four exons and three introns within the coding region. The two middle exons were almost identical in size, reflecting the conservation of the central domain. The first and fourth exons demonstrated more variability, while the size of the three introns varied greatly. Type C genes have five exons, the first four of which are similar to the exons of type A and B genes, while the additional fifth exon encodes most of the cysteine-rich tail. Projection of exon boundaries on the protein sequence displayed identical positions for all tested plant Gγ genes. Noticeably, Gγ genes from other eukaryotes differ from those of plants and each other in relation to intron numbers and positions. Gγ genes from some fungi (*Saccharomyces cerevisiae, Ashbya gossypii*), the primitive eukaryote *Naegleria gruberi* and some metazoans such as several species of *Drosophila* and sea anemone *Nematostella vectensis* do not have introns (data not shown). On the other hand, stramenopiles (*Phytophthora infestans*, *Ectocarpus siliculosus*), fungi (*Aspergillus clavatus, Laccaria bicolor*), the social amoeba *Dictyostelium discoideum* and most animals have 1–3 introns in variable positions (Additional file 1: Figure S3). All functional human Gγ genes have one intron in the same position [25]. Two human genes are presented in Additional file 1: Figure S3. At the same time several species from taxa related to vertebrates (such as Hemichordata, Cephalochordata and Echinodermata) have Gγ genes with and without introns. These observations provide additional evidence supporting the close relationship within plant Gγ genes, while demonstrate that exon-intron arrangements in other eukaryotic Gγ genes were variable even within closely related groups. Importantly, one intron position (projected to be approximately in the middle of the protein sequence) was common for the majority of Gγ genes with introns (Additional file 1: Figure S3), uniting an incredibly diverse range of eukaryotes, including plants, animals, fungi, amoebazoans and stramenopiles.

### C-terminus variability in eukaryotic Gγ subunits

The presence of a CaaX box and specifically the cysteine residue in the fourth position from the protein end, providing a possibility for protein prenylation, is considered a prominent feature of all known Gγ subunits [1,29,45-47]. However, we found that plant Gγ subunits often lack the canonical prenylation motif. Only type A, invariably has a CaaX motif, while type B totally lacks cysteines at the C-terminus and only approximately half of the available type C proteins contain a CaaX-like motif.

To study the C-termini of Gγ subunits from different eukaryotes we screened the available sequence databases using a number of established Gγ sequences as a query for BLAST searches. In total ~150 Gγ subunits from several eukaryotic groups were compared. For each eukaryotic group a consensus of ~20 C-terminal amino acids was obtained using the majority rule, when ‘majority’ is determined by over 50% presence; highly variable positions were substituted with ‘x’ (Table 1). To avoid over-representation of a single group, such as vertebrates in the consensus for animals, we first produced preliminary consensus sequences for all phyla and then joined them producing the final consensus for the kingdom. The results reflect an abundance of valine, isoleucine, methionine and leucine in the two terminal positions, while third position from the end was found to be variable in all eukaryotes, except for plants, where it is predominantly occupied by tryptophan (Table 1). Interestingly, we found that one of the most primitive single cell eukaryote, *Naegleria gruberi*, has two Gγ subunits. One of them contains classical CaaX box, while the second protein has no prenylation motif. Unlike in plants, where non-prenylated Gγ subunits are an abundant type, Naegleria’s non-prenylated variant is an isolated case. It has the highest sequence similarity with its paralog containing the CaaX motif, suggesting relatively recent divergence of the two proteins.

**Table 1:** C-terminal amino acids of Gγ subunits from different eukaryotes

**Species / Group**	**C-terminal amino acids**
Animals	VxxPxSENPFKEKKxG**C**xIL
Fungi	VDKxEDPYAPxxxGGC**C**xVM
Amoebozoa	FxGExNPWxxNQxGGC**C**xVI
Stramenopiles	NDxPNxWQQSxQGGGG**C**xIL
Plants type A	xWDRWFEGPQDxxGCR**C**WIL
*Naegleria gruberi* FE249419	SDPTNPYLNPPKDGGC**C**MIM

## Discussion

The importance of heterotrimeric G-proteins in plant cell signalling is difficult to overestimate, being involved in multiple developmental processes [5,8-10,14,48-50] and innate immunity [6,7,51-56]. Most of the available studies have used Arabidopsis and rice mutants lacking either Gα or Gβ, each encoded by a single gene in these species. In contrast, Gγ is encoded by several genes in both species and their important role for the correct function of the heterotrimer has been recently demonstrated [10,14,15,29,37]. In this study we have analysed over 200 Gγ subunits from nearly 100 seed bearing plant species and found striking differences in their C-termini with many subunits lacking the ‘consensus’ C-terminal prenylation motif, CaaX.

### Prenylated and non-prenylated plant Gγ subunits

It has been established in animal studies and also demonstrated in plants, that Gγ anchors the Gβ subunit to the plasma membrane [1,3,10-12,21,22,27,28,32]. This is achieved by strongly binding Gβ using a coiled-coil interaction and targeting the dimer to the plasma membrane via post-translational modification of the Gγ C-terminus. Classically, post-translational processing of the Gγ subunits includes prenylation and proteolytic cleavage of the last three amino acids [30-32]. Prenylation of protein C-termini in eukaryotes is catalysed by three types of protein prenyltransferases. Protein farnesyltransferase (PFT) transfers a farnesyl group to the cysteine residue of a CaaX motif, where “a” is preferably an aliphatic amino acid and “X” is preferably, but not necessarily, methionine, glutamine, alanine, cysteine, or serine. Protein geranylgeranyltransferase type I (PGGT I) catalyses the transfer of a geranylgeranyl moiety to the cysteine residue of a CaaX motif, where “X” is preferably leucine or isoleucine. Finally, protein geranylgeranyltransferase type II (PGGT II) is highly specific for RAB-GTPases and requires an escort protein, therefore, it does not participate in prenylation of Gγ subunits [57,58]. Thus, the presence of the CaaX motif is considered to be compulsory for Gγ subunits.

We found that in plants only type A and about half of type C Gγ subunits possess a putative CaaX motif, while type B proteins do not have it. Even though the plant CaaX motif is similar to that of other eukaryotes in general terms, it has a distinct difference. In plants, the first aliphatic amino acid position of the CaaX box is frequently occupied with an aromatic amino acid tryptophan, which is otherwise never found in this position or even in the proximity of the prenylated cysteine in any other eukaryotes. This deviation from the mammalian pattern raises questions about the possible prenylation of those plant Gγ subunits containing a CWIL motif and the ability of non-prenylated types (B and C) to provide membrane localization for the Gβγ dimer.

It has been reported that two of the Arabidopsis Gγ subunits, AGG1 and AGG2, are prenylated *in vivo*[27,28]. Both proteins are type A subunits and contain conventional CaaX motifs, (CLIL and CSIL respectively), being rather exceptions to the type A consensus C-termini (CWIL). On the other hand, since the first aliphatic amino acid is solvent exposed and is not involved in any hydrophobic interactions, as are the two terminal amino acids, its position in CaaX could in theory accommodate any amino acid [59]. This theoretical rule is supported by the high variability observed in this position in all eukaryotes (Table 1). In fact, a human Gγ5 subunit with an artificially added CWIL C-terminal motif was prenylated in transfected cells, but proteolytic cleavage of the three terminal amino acids was suppressed [60]. Therefore it is possible that despite the presence of tryptophan in place of the first aliphatic residue in CaaX plant Gγ subunits, they could still be prenylated. Interestingly, it has been recently established that Arabidopsis PGGT I exhibits more specificity for CaaX motifs containing leucine in the terminal position than PGGT I enzymes from metazoans and yeast [61]. We found that most type A subunits terminate in leucine (Figure 1B, Table 1). This may imply that type A Gγ subunits are mostly modified with a geranylgeranyl moiety. The preferential prenylation of Arabidopsis AGG1 and AGG2 subunits by PGGT I has been experimentally demonstrated [27,28]. On the other hand, it was shown that Arabidopsis AGG2 that ends in CGCSIL is prenylated on the −4 cysteine (4th position from the end) while is also S-acylated on the −6 cysteine, demonstrating a dual lipidation [27,28]. This feature, two cysteines separated by an amino acid, is conserved in type A sequences (Figure 1B, Table 1), suggesting that S-acylation of the −6 cysteine could be a common modification for plant Gγ subunits. The presence of aromatic and basic amino acids has been confirmed to promote S-acylation of a nearby cysteine [62,63]. Therefore, the tryptophan present in the most common C-terminus of type A (CRCWIL) could serve as a facilitator of S-acylation of the −6 cysteine, assuming that it is not cleaved after prenylation of the second cysteine.

The plant Gγ subunits without prenylation motifs, namely type B and half of type C subunits, cannot be prenylated by PFT or PGGT I. Nevertheless, they can still be functional Gγ subunits anchoring the Gβγ dimer to the membrane. A prominent characteristic of all these sequences is an overrepresentation of positively charged amino acids (arginine, lysine) combined with aromatic residues (tryptophan, phenylalanine) and highly hydrophobic residues (isoleucine and leucine) at the C-terminus. Such amino acid combination is also found at the C-terminus of a number of membrane proteins and it has been established that these amino acids form an amphipathic α-helix, able to anchor the proteins to the plasma membrane [64-68]. We therefore hypothesize that a similar structure could be formed at the C-terminus of plant Gγ subunits providing affinity for the plasma membrane. A representative of type B, rice RGG2, was detected in the plasma membrane fraction, despite absence of a prenylation motif [26]. At the same time YFP-fused type B subunits from soybean were also detected on the plasma membrane [19]. Additionally, in the Arabidopsis mutant *plp* lacking the common PFT/PGGT I α-subunit and, hence, incapable of prenylation, AGG2 was shown to form stable membrane association, assumingly due to a polybasic C-terminal block [28]. No doubt, further experimental data is required to unequivocally establish the intracellular topology of the type B Gγ subunits and post-translational modification patterns of plant Gγ subunits in general. Nevertheless, we would like to draw attention to the possibility that plant’s unique C-terminal structure enables Gγ subunits to target the plasma membrane regardless of the prenylation status, which would greatly distinguish them from their animal counterparts.

### A new nomenclature for plant Gγ subunits

We believe that it will be very advantageous in the future to unequivocally identify the different Gγ subunits as members of one of the three types defined in this report, since it could point to specific post-translational protein processing and possibly have important functional implications. For this reason we propose to use the type as an integral part of the Gγ subunits’ name for future studies. We realize the limits of our analysis and predict that new types of Gγ subunits could be discovered in the future. For instance our preliminary studies revealed a new form (type?) of Gγ related proteins in primitive land plants (Trusov, Botella unpublished data). Therefore, we emphasize that the new proposed nomenclature will easily accommodate newly discovered types, by using the sequential letter code (A, B, C, D, E and so on). It is also important to consider the common directions for plant nomenclature widely accepted by plant biologists to use the two first letters to represent a species name. In this way the three existing Arabidopsis Gγ subunits could be referred as “AtGGA1”, referring to *Arabidopsisthaliana*G-protein Gamma subunit type A, number 1, (currently named “AGG1” [11]), “AtGGA2” (currently named “AGG2” [12]) and “AtGGC1” (currently named “AGG3”[10]). Similarly, for rice the nomenclature would apply as “OsGGA1” (currently named “RGG1” [26]), “OsGGB1” (currently named “RGG2” [26]), “OsGGC1” (currently named “GS3” [34]), “OsGGC2” (AACV01018633.1 ) and “OsGGC3” (currently named “DEP1” [35]).

## Conclusion

We would like to make a short note to propose some caution on how to interpret past and future functional research into the Arabidopsis AtGGA1 and AtGGA2 subunits (previously AGG1 and AGG2). It is important to keep in mind that both subunits belong to type A and that no type B subunit is present in Arabidopsis; therefore generalizations to other plants species are inherently risky. It is possible that AtGGA1 and AtGGA2 have evolved to take up some or all of the functions that type B subunits have in other species. It is thus important to study type A subunits in other species since they could have different or more restricted roles than those observed for AtGGA1 and AtGGA2. In addition, it is also important to establish the role of type B subunits, for which we still don’t have any functional information.

## Methods

### Database searches

BLAST searches against the Genebank databases were run using full-length AGG1 (AF283673), AGG2 (AF347077), AGG3 (AAT85756), RGG1 (AB120662) and RGG2 (AP005647) protein sequences known to be plant Gγ subunits. The soybean proteins [19] were published later than our original search was done. From the significant hits a subset of approximately 500 sequences was extracted, covering over 100 land plants species. The low score sequences were also considered and analyzed if conserved motifs (DPLL/I and CaaX* (asterisk represent the termination signal)) were identified. ESTs presenting parts of a putative gene were assembled by eye using SED.exe program from VOSTORG package [69]. Sequences containing identical or almost identical reading frames were considered redundant and reduced to one. Sequences containing full length or nearly full length reading frames were selected for further analysis.

### Phylogenetic analysis

Protein sequence alignment was done using CLUSTAL-OMEGA [40]. The program is provided on EMBL-EBI web site http://www.ebi.ac.uk. Manual alignments were done using program SED.exe and analysis of inversions and repeats was done with dotmap.exe provided in package VOSTORG [69].

Phylogenetic analysis was performed using package PHYLIP 3.66 [41]. The package was used as described in manual provided with the programs. Namely, the seqboot.exe with default settings was used to generate file with 1000 bootstrapped data sets. 100 bootstraps were used for preliminary analyses to generate phylogenetic trees with the following algorithms: Protein/nucleotide Sequence Parsimony, Protein/nucleotide maximum likelihood, Neighbor-Joining, UPGMA, Fitch-Margoliash. All programs were used at default settings except for resetting for multiple data sets. 1000 bootstraps were generated for final parsimonious trees reconstructions. Package PhyML-aBayes [42,43] was also used to generate maximum likelihood tree using different substitution models for stationary amino-acid frequencies provided with the program.

Protein weblogos for Figure 1B were designed using Berkley University website http://weblogo.berkeley.edu/logo.cgi[70].

Phylogenetic trees were visualized using program FigTree v1.3.1. http://tree.bio.ed.ac.uk/software/figtree/.

### Availability of supporting data

The additional data supporting the results of this article is available from authors by request.

## Competing interests

The authors declare that they have no competing interests.

## Authors’ contributions

YT collected data, conducted phylogenetic, structural and statistical analyses. DC was involved in data collection, interpretation of the results and has made substantial contributions to conception and design of the project. JB designed and coordinated the project. YT and JB wrote the manuscript. All the authors read and approved the final manuscript.

## Supplementary Material

Additional file 1 **Figure S1.** Repetitive elements in *Gossypium raimondii* putative Gγ type C subunit sequence. The elements are highlighted in yellow/green. **Figure S2.** Insertions/deletions in Gγ type C gene. * indicates identical nucleotides. **Figure S3**. Projection of exon boundaries on protein sequence of GγC subunits from selected eukaryotic species; conserved DPLL motif highlighted in green; hyphen and amino acids highlighted in yellow display positions of exon boundaries. **Table S1.** Amino acid content (%) and approximate length of the cysteine-rich domain of type C Gγ subunits. Click here for file

## References

[B1] Gautam N Downes GB Yan K Kisselev O The G-protein βγ complex Cell Signal 1998 10 447 455 10.1016/S0898-6568(98)00006-0 9754712

[B2] Gilman AG G-proteins - transducers of receptor-generated signals Annu Rev Biochem 1987 56 615 649 10.1146/annurev.bi.56.070187.003151 3113327

[B3] McIntire WE Structural determinants involved in the formation and activation of G protein βγ dimers Neurosignals 2009 17 82 99 10.1159/000186692 19212142 PMC2836951

[B4] Assmann SM Plant G proteins, phytohormones, and plasticity: three questions and a speculation Science's STKE 2004 2004 20 10.1126/stke.2642004re20 15613689

[B5] Perfus-Barbeoch L Jones AM Assmann SM Plant heterotrimeric G protein function: insights from arabidopsis and rice mutants Current Opinion in Plant Biology 2004 7 719 731 10.1016/j.pbi.2004.09.013 15491922

[B6] Trusov Y Botella JR New faces in plant innate immunity: heterotrimeric G proteins J Plant Biochem Biotechnol 2012 in press

[B7] Trusov Y Jorda L Molina A Botella JR G proteins and plant innate immunity 2010 Netherlands: Springer

[B8] Warpeha KM Upadhyay S Yeh J Adamiak J Hawkins SI Lapik YR Anderson MB Kaufman LS The GCR1, GPA1, PRN1, NF-Y signal chain mediates both blue light and abscisic acid responses in arabidopsis Plant Physiol 2007 143 1590 1600 10.1104/pp.106.089904 17322342 PMC1851835

[B9] Assmann SM Heterotrimeric and unconventional GTP binding proteins in plant cell signalling Plant Cell 2002 2002 S355 373 Supplement 12045288 10.1105/tpc.001792 PMC151266

[B10] Chakravorty D Trusov Y Zhang W Sheahan MB Acharya BW McCurdy DW Assmann SM Botella JR A highly atypical heterotrimeric G protein γ subunit is involved in guard cell K+ channel regulation and morphological development in Arabidopsis thaliana Plant J 2011 67 840 851 10.1111/j.1365-313X.2011.04638.x 21575088

[B11] Mason MG Botella JR Completing the heterotrimer: isolation and characterization of an arabidopsis thaliana G protein γ -subunit cDNA Proc Natl Acad Sci U S A 2000 97 14784 14788 10.1073/pnas.97.26.14784 11121078 PMC18996

[B12] Mason MG Botella JR Isolation of a novel G-protein γ-subunit from arabidopsis thaliana and its interaction with Gβ Biochim Biophys Acta 2001 1520 147 153 10.1016/S0167-4781(01)00262-7 11513956

[B13] Thung L Trusov Y Chakravorty D Botella JR Gγ1+Gγ2+Gγ3=Gβ: the search for heterotrimeric G-protein γ subunits in arabidopsis is over J Plant Physiology 2012 169 542 545 10.1016/j.jplph.2011.11.010 22209167

[B14] Trusov Y Rookes JE Tilbrook K Chakravorty D Mason MG Anderson D Chen JG Jones AM Botella JR Heterotrimeric G protein γ subunits provide functional selectivity in G βγ dimer signaling in arabidopsis Plant Cell 2007 19 1235 1250 10.1105/tpc.107.050096 17468261 PMC1913745

[B15] Trusov Y Zhang W Assmann SM Botella JR Gγ1+Gγ2 ≠ Gβ: heterotrimeric G protein Gγ-deficient mutants do not recapitulate all phenotypes of Gβ-deficient mutants Plant Physiol 2008 147 636 649 10.1104/pp.108.117655 18441222 PMC2409028

[B16] Bisht NC Jez JM Pandey S An elaborate heterotrimeric G-protein family from soybean expands the diversity of plant G-protein networks New Phytol 2010 190 35 48 10.1111/j.1469-8137.2010.03581.x 21175635

[B17] Hossain MS Koba T Harada K Cloning and characterization of two full-length cDNAs, TaGA1 and TaGA2, encoding G-protein α subunits expressed differentially in wheat genome Genes & Genetic Systems 2003 78 127 138 10.1266/ggs.78.127 12773813

[B18] Misra S Wu Y Venkataraman G Sopory SK Tuteja N Heterotrimeric G-protein complex and G-protein-coupled receptor from a legume (Pisum sativum): role in salinity and heat stress and cross-talk with phospholipase C Plant J 2007 51 656 669 10.1111/j.1365-313X.2007.03169.x 17587233

[B19] Choudhury SR Bisht NC Thompson R Todorov O Pandey S Conventional and novel Gγ protein families constitute the heterotrimeric G-protein signaling network in soybean PLoS One 2011 6 e23361 10.1371/journal.pone.0023361 21853116 PMC3154445

[B20] Lambright DG Sondek J Bohm A Skiba NP Hamm HE Sigler PB The 2.0 A crystal structure of a heterotrimeric G protein Nature 1996 379 311 319 10.1038/379311a0 8552184

[B21] Sondek J Bohm A Lambright DG Hamm HE Sigler PB Crystal structure of a G-protein By dimer at 2.1A resolution Nature 1996 379 369 374 10.1038/379369a0 8552196

[B22] Marrari Y Crouthamel M Irannejad R Wedegaertner PB Assembly and trafficking of heterotrimeric G proteins Biochemistry 2007 46 7665 7677 10.1021/bi700338m 17559193 PMC2527407

[B23] Anderson DJ Botella JR Expression analysis and subcellular localization of the Arabidopsis thaliana G-protein β-subunit AGB1 Plant Cell Reporter 2007 26 1469 1480 10.1007/s00299-007-0356-1 17492287

[B24] Balcueva EA Wang Q Hughes H Kunsch C Yu Z Robishaw JD Human G protein γ11 and γ14 subtypes define a new functional subclass Experimental Cell Res 2000 257 310 319 10.1006/excr.2000.4893 10837145

[B25] Hurowitz EH Melnyk JM Chen YJ Kouros-Mehr H Simon MI Shizuya H Genomic characterization of the human heterotrimeric G protein α, β, and γ subunit genes DNA Res 2000 7 111 120 10.1093/dnares/7.2.111 10819326

[B26] Kato C Mizutani T Tamaki H Kumagai H Kamiya T Hirobe A Fujisawa Y Kato H Iwasaki Y Characterisation of heterotrimeric G protein complexs in rice plasma membrane Plant J 2004 38 320 331 10.1111/j.1365-313X.2004.02046.x 15078334

[B27] Adjobo-Hermans MJW Goedhart J Gadella TWJ Jr Plant G protein heterotrimers require dual lipidation motifs of Gα and Gγ and do not dissociate upon activation J Cell Sci 2006 119 5087 5097 10.1242/jcs.03284 17158913

[B28] Zeng Q Wang X Running MP Dual lipid modification of Arabidopsis Gγ-subunits is required for efficient plasma membrane targeting Plant Physiol 2007 143 1119 1131 10.1104/pp.106.093583 17220359 PMC1820929

[B29] Chakravorty D Botella JR Over-expression of a truncated Arabidopsis thaliana heterotrimeric G protein γ subunit results in a phenotype similar to α and β subunit knockouts Gene 2007 393 163 170 10.1016/j.gene.2007.02.008 17383830

[B30] Iniguez-Lluhi JA Simon MI Robishaw JD Gilman AG G protein by subunits synthesized in Sf9 cells. Functional characterization and the significance of prenylation of y J Biol Chem 1992 267 23409 23417 1429682

[B31] Simonds WF Butrynski JE Gautam N Unson CG Spiegel AM G-protein βγ dimers. Membrane targeting requires subunit coexpression and intact γ C-A-A-X domain J Biol Chem 1991 266 5363 5366 1706334

[B32] Takida S Wedegaertner PB Heterotrimer formation, together with isoprenylation, is required for plasma membrane targeting of Gβγ J Biol Chem 2003 278 17284 17290 10.1074/jbc.M213239200 12609996

[B33] Altschul SF Koonin EV Iterated profile searches with PSI-BLAST–a tool for discovery in protein databases Trends in Biochemical Sci 1998 23 444 447 10.1016/S0968-0004(98)01298-5 9852764

[B34] Fan C Xing Y Mao H Lu T Han B Xu C Li X Zhang Q GS3, a major QTL for grain length and weight and minor QTL for grain width and thickness in rice, encodes a putative transmembrane protein Theor Appl Genet 2006 112 1164 1171 10.1007/s00122-006-0218-1 16453132

[B35] Huang X Qian Q Liu Z Sun H He S Luo D Xia G Chu C Li J Fu X Natural variation at the DEP1 locus enhances grain yield in rice Nat Genet 2009 41 494 497 10.1038/ng.352 19305410

[B36] Takano-Kai N Jiang H Kubo T Sweeney M Matsumoto T Kanamori H Padhukasahasram B Bustamante C Yoshimura A Doi K McCouch S Evolutionary history of GS3, a gene conferring grain length in rice Genetics 2009 182 1323 1334 10.1534/genetics.109.103002 19506305 PMC2728869

[B37] Botella JR Can heterotrimeric G proteins help to feed the world? Trends Plant Sci 2012 17 563 568 10.1016/j.tplants.2012.06.002 22748359

[B38] Cook LA Schey KL Cleator JH Wilcox MD Dingus J Hildebrandt JD Identification of a region in G protein γ subunits conserved across species but hypervariable among subunit isoforms Protein Sci 2001 10 2548 2555 11714923 10.1110/ps.ps.26401 PMC2374038

[B39] Temple BRS Jones AM The plant heterotrimeric G-protein complex Annual Rev Plant Biology 2007 58 249 266 10.1146/annurev.arplant.58.032806.103827 17201690

[B40] Sievers F Wilm A Dineen D Gibson TJ Karplus K Li W Lopez R McWilliam H Remmert M Soding J Fast, scalable generation of high-quality protein multiple sequence alignments using Clustal Omega Mol Syst Biol 2011 7 539 21988835 10.1038/msb.2011.75 PMC3261699

[B41] Felsenstein J PHYLIP - phylogeny inference package (version 3.2) Cladistics 1989 5 164 166

[B42] Guindon S Dufayard JF Lefort V Anisimova M Hordijk W Gascuel O New algorithms and methods to estimate maximum-likelihood phylogenies: assessing the performance of PhyML 3.0 Syst Biol 2010 59 307 321 10.1093/sysbio/syq010 20525638

[B43] Guindon S Gascuel O A simple, fast, and accurate algorithm to estimate large phylogenies by maximum likelihood Syst Biol 2003 52 696 704 10.1080/10635150390235520 14530136

[B44] Finet C Timme RE Delwiche CF Marletaz F Multigene phylogeny of the green lineage reveals the origin and diversification of land plants Curr Biol 2010 20 2217 2222 10.1016/j.cub.2010.11.035 21145743

[B45] Jansen G Thijssen KL Werner P van der Horst M Hazendonk E Plasterk RH The complete family of genes encoding G proteins of caenorhabditis elegans Nat Genet 1999 21 414 419 10.1038/7753 10192394

[B46] Krystofova S Borkovich KA The heterotrimeric G-protein subunits GNG-1 and GNB-1 form a Gβγ dimer required for normal female fertility, asexual development, and galpha protein levels in Neurospora crassa Eukaryotic Cell 2005 4 365 378 10.1128/EC.4.2.365-378.2005 15701799 PMC549333

[B47] Zhang N Long Y Devreotes PN Gγ in Dictyostelium: its role in localization of Gβγ to the membrane is required for chemotaxis in shallow gradients Mol Biology of the Cell 2001 12 3204 3213 10.1091/mbc.12.10.3204 PMC60167 11598203

[B48] Jones AM Assmann SM Plants: the latest model system for G-protein research EMBO Rep 2004 5 572 578 10.1038/sj.embor.7400174 15170476 PMC1299082

[B49] Ullah H Chen J-G Temple B Boyes DC Alonso JM Davis KR Ecker JR Jones AM The β-Subunit of the Arabidopsis G protein negatively regulates auxin-induced cell division and affects multiple developmental processes Plant Cell 2003 15 393 409 10.1105/tpc.006148 12566580 PMC141209

[B50] Warpeha KM Kaufman LS Opposite ends of the spectrum: plant and animal g-protein signaling Plant Signaling and Behavior 2007 2 480 482 10.4161/psb.2.6.4497 19704591 PMC2634341

[B51] Llorente F Alonso-Blanco C Sanchez-Rodriguez C Jorda L Molina A ERECTA receptor-like kinase and heterotrimeric G protein from arabidopsis are required for resistance to the necrotrophic fungus plectosphaerella cucumerina Plant J 2005 43 165 180 10.1111/j.1365-313X.2005.02440.x 15998304

[B52] Suharsono U Fujisawa Y Kawasaki T Iwasaki Y Satoh H Shimamoto K The heterotrimeric G protein α subunit acts upstream of the small GTPase Rac in disease resistance of rice Proc Natl Acad Sci U S A 2002 99 13307 13312 10.1073/pnas.192244099 12237405 PMC130629

[B53] Trusov Y Rookes JE Chakravorty D Armour D Schenk PM Botella JR Heterotrimeric G-proteins facilitate arabidopsis resistance to necrotrophic pathogens and are involved in jasmonate signaling Plant Physiol 2006 140 210 220 16339801 10.1104/pp.105.069625 PMC1326045

[B54] Trusov Y Sewelam N Rookes JE Kunkel M Nowak E Schenk PM Botella JR Heterotrimeric G proteins-mediated resistance to necrotrophic pathogens includes mechanisms independent of salicylic acid-, jasmonic acid/ethylene- and abscisic acid-mediated defense signaling Plant J 2009 58 69 81 10.1111/j.1365-313X.2008.03755.x 19054360

[B55] Zhang W He SY Assmann SM The plant innate immunity response in stomatal guard cells invokes G-protein-dependent ion channel regulation Plant J 2008 56 984 996 10.1111/j.1365-313X.2008.03657.x 18702674 PMC2804871

[B56] Delgado-Cerezo M Sanchez-Rodriguez C Escudero V Miedes E Fernandez PV Jorda L Hernandez-Blanco C Sanchez-Vallet A Bednarek P Schulze-Lefert P Arabidopsis heterotrimeric G-protein regulates cell wall defense and resistance to necrotrophic fungi Mol Plant 2012 5 98 114 10.1093/mp/ssr082 21980142

[B57] Crowell DN Huizinga DH Protein isoprenylation: the fat of the matter Trends Plant Sci 2009 14 163 170 10.1016/j.tplants.2008.12.001 19201644

[B58] Maurer-Stroh S Washietl S Eisenhaber F Protein prenyltransferases Genome Biol 2003 4 212 10.1186/gb-2003-4-4-212 12702202 PMC154572

[B59] Lane KT Beese LS Thematic review series: lipid posttranslational modifications. Structural biology of protein farnesyltransferase and geranylgeranyltransferase type I J Lipid Res 2006 47 681 699 10.1194/jlr.R600002-JLR200 16477080

[B60] Kilpatrick EL Hildebrandt JD Sequence dependence and differential expression of Gγ5 subunit isoforms of the heterotrimeric G proteins variably processed after prenylation in mammalian cells J Biol Chem 2007 282 14038 14047 10.1074/jbc.M701338200 17353195

[B61] Andrews M Huizinga DH Crowell DN The CaaX specificities of Arabidopsis protein prenyltransferases explain era1 and ggb phenotypes BMC Plant Biology 2010 10 118 10.1186/1471-2229-10-118 20565889 PMC3017772

[B62] Bizzozero OA Bixler HA Pastuszyn A Structural determinants influencing the reaction of cysteine-containing peptides with palmitoyl-coenzyme A and other thioesters Biochim Biophys Acta 2001 1545 278 288 10.1016/S0167-4838(00)00291-0 11342053

[B63] Hemsley PA Protein S-acylation in plants Mol Membr Biol 2009 26 114 125 10.1080/09687680802680090 19191173

[B64] Gambhir A Hangyas-Mihalyne G Zaitseva I Cafiso DS Wang J Murray D Pentyala SN Smith SO McLaughlin S Electrostatic sequestration of PIP2 on phospholipid membranes by basic/aromatic regions of proteins Biophys J 2004 86 2188 2207 10.1016/S0006-3495(04)74278-2 15041659 PMC1304070

[B65] Prinz WA Hinshaw JE Membrane-bending proteins Crit Rev Biochem Mol Biol 2009 44 278 291 10.1080/10409230903183472 19780639 PMC3490495

[B66] Zhang W Crocker E McLaughlin S Smith SO Binding of peptides with basic and aromatic residues to bilayer membranes: phenylalanine in the myristoylated alanine-rich C kinase substrate effector domain penetrates into the hydrophobic core of the bilayer J Biol Chem 2003 278 21459 21466 10.1074/jbc.M301652200 12670959

[B67] Roy MO Leventis R Silvius JR Mutational and biochemical analysis of plasma membrane targeting mediated by the farnesylated, polybasic carboxy terminus of K-ras4B Biochemistry 2000 39 8298 8307 10.1021/bi000512q 10889039

[B68] Yeung T Terebiznik M Yu L Silvius J Abidi WM Philips M Levine T Kapus A Grinstein S Receptor activation alters inner surface potential during phagocytosis Science 2006 313 347 351 10.1126/science.1129551 16857939

[B69] Zharkikh AA Rzhetsky A Morosov PS Sitnikova TL Krushkal JS VOSTORG: a package of microcomputer programs for sequence analysis and construction of phylogenetic trees Gene 1991 101 251 254 10.1016/0378-1119(91)90419-C 2055489

[B70] Crooks GE Hon G Chandonia JM Brenner SE WebLogo: a sequence logo generator Genome Res 2004 14 1188 1190 10.1101/gr.849004 15173120 PMC419797

