# Supplementary material for: Diversity of heterotrimeric G-protein γ subunits in plants
Source: BMC Res Notes. 2012 Oct 31;5:608. doi: 10.1186/1756-0500-5-608 (PMC3508898; doi:10.1186/1756-0500-5-608)
Supplement: Additional file 1 — Figure S1. Repetitive elements in Gossypium raimondii putative Gγ type C subunit sequence. The elements are highlighted in yellow/green. Figure S2. Insertions/deletions in Gγ type C gene. * indicates identical nucleotides. Figure S3. Projection of exon boundaries on protein sequence of GγC subunits from selected eukaryotic species; conserved DPLL motif highlighted in green; hyphen and amino acids highlighted in yellow display positions of exon boundaries. Table S1. Amino acid content (%) and approximate length of the cysteine-rich domain of type C Gγ subunits. [file 1756-0500-5-608-S1.pdf]

Figure S1. Repetitive elements in *Gossypium raimondii* putative Gγ type C subunit sequence. The elements are highlighted in yellow/green. The alignment of the elements is shown below;
* indicates identical amino acids

```
MAARSGGSSSSSVPTLPPPCPRSPPEYPDLYGKRREAAKTQMLEREISFLEEELKSVEGLQPASRFCKEVTDFVMANSDPLIPTSRKNRKSRRFWKWLCGIPCFNLSWICCCCYSACSCHLKCLQCSGLQCCDCDCDLCNCSSCICSLCKCRCCSCGSSEHSSCKCSSCDLPSCKCSSCNCTSCKCSSCNCNPCKCSSCDCNPCKCSSCNCNPCKCSSCDCSSCKCSSLCDCSSCKCSSLCDCSSCKCSSCNCSSCKCSSLCDCSSCKCSSCDCKSCLSCCTIPKWRCKCSSCNPCHCNPCLSCCSILKWRCCSFPKSGCCKKISCSRNCCIFQFPSCTGCFCCKWKCSCPKVTRCCSYTKTSCNPCCLFF*

EHSSCKCSS-C
DLPSCKCSS-C
NCTSCKCSS-C
NCNPCKCSS-C
DCNPCKCSS-C
NCNPCKCSS-C
DCSSCKCSSLC
DCSSCKCSSLC
DCSSCKCSS-C
NCSSCKCSSLC
DCSSCKCSS-C
    ***** *
```

Figure S2. Insertions/deletions in Gγ type C gene. * indicates identical nucleotides.

```
A.thaliana      ATGTCTGCTCCTTCTGGCGGTGGCGAAGGAGGAGGAAAAGAATCAGCTGCTGGTGGAGTG 60
B.rapa          ATGGCTTCTACTTCATGCAGTGTCAAAGTA------------------GCTGGTGAAACA 42
                *** ** ** ****  ** *** * *** *                  ******* *

A.thaliana      AGTTCATCGTCTCTTGCTCCGTCGTCTCTACCACCGCCTCGTCCTAAGTCTCCACCAGAG 120
B.rapa          GGT---TTGGCTCTTGCTAAGTCATCTCTACCGCCCCCTCGTCCCAAGTCTCCGCCGCCG 99
                 **   * * ********  *** ******** ** ******** ******* **   *

A.thaliana      TATCCAGATTTGTACGGGAAACGCAGAGAGGCGGCGAGAGTTCAGATGCTCGAGAGAGAG 180
B.rapa          TATCCAGATTTGTACGGGAAACGTAGAGAGGCGGCGAGAGTTCAGATGCTAGAGAGAGAG 159
                *********************** ************************* *********

A.thaliana      ATTGGTTTTCTCGAGGGCGAAATTAAATTCATCGAAGGCGTACAACCGGCATCTAGATGC 240
B.rapa          ATTGGTTTTCTCGAGGGAGAAATTAAATTTGTCGAAGGCTTGCAACCGTCGTCCAGATGC 219
                **************** ***********  ******** * ****** * ** ******

A.thaliana      ATCAAAGAAGTCTCTGATTTTGTTGTTGCAAATTCTGACCCATTGATCCCTGCACAACGA 300
B.rapa          TGCAAAGAGGTCTCTGATTTTGTGGTTGCAAATTCTGACCCAATGATACCTGCACAACGA 279
                  ****** ************** ***************** **** ************

A.thaliana      AAAAGTCGAAGATCCTTCCGGTTCTGGAAGTGGCTCTGTGGCCCATGTTTGAGCCTGGTG 360
B.rapa          AAGAGTCGACGACACTGCCGGTTCTGGAAGTGGCTCTGTTTCCCGTGTTTGAGCGTGGCG 339
                ** ****** **  ** *********************  *** ********* *** *

A.thaliana      AGTTTCTGCTGTTGCTGCCAATCCAAATGTTCGTGCCATCTGAGGAAACCCAAGTGCTGC 420
B.rapa          AGTTTCTGCTGTTGTTGCCGATCCGAGTGTTCGTGCCATCTGAGGAAGCCAAAATGCTGC 399
                ************* **** **** * ******************* ** ** ******

A.thaliana      AACTGTACATCTTGCAGCTGTATAGGGTCCAAATGCTGTGACGGGTCATGCTGCTCAAAC 480
B.rapa          AACTCCACATGCTGTAGCTGCGTTGGTTCCAAATGCTTCAACGGGTCCTGCTGCTCAAAC 459
                ****  ****  ** *****  * ** **********   ******* ***********

A.thaliana      ATTTGTTGTTGCCCGAGA------------------CTAAGCTGCCCGAGCTGTTCATGC 483
B.rapa          AGCTGTTGTTGCCCGAAACCGAGCTGCCCCAAATGCCTGAGCTGCCCGAGCTGTTCATGC 519
                *  ************* *                  ** *******************

A.thaliana      TTCCGAGGTTGCTGGTGT------------------------------------------ 531
B.rapa          TTCCGAGGTTGCTGTTGTTGCCCGAAACCGAGCTGCCCGAGCTGTTCATGCTTCCGAGGT 579
                ************** ***

A.thaliana      ---------TCTTGTCCGGACATGTCTTGCTGCATTCCCAGCTGTTTCCGCAGTTGCAGT 591
B.rapa          TGCTGTTGTTCTTGTCCGGACTTATGTTGTTGCATACCCACCTGTTTCCGCAGTTGCAG- 638
                         *********** * * *** ***** **** *****************

A.thaliana      TGCACTCGACCGTCGTGTCTGAATAAAAAGAAGAGCTCATGCTGCAGCTGCAACTGCAA- 650
B.rapa          -----TCGACCATCATGTCT---TAATAAGAAGAGCTCATGTTGCAGCTGCAACTGCAAC 690
                     ****** ** *****   *** ************** ****************

A.thaliana      -----GATCAGATGGTCATCTTGTTTTAGTTGTCCCAAGGTACGACTTTGTTCTTGTTGT 705
B.rapa          TGCAAGATCAAATGGTCATCTTGTTTTAAATGTCCTAAGCTACGACTTTGT---TGTTGT 747
                     ***** ****************  ***** *** ***********   ******

A.thaliana      TTTTGCAATTGTAAAAATCTATGTTCTAATCCTTGTTGTTTAGCTTTCTAA 756
B.rapa          TTTTGCAATTGTAAAAATACATGTTCTTATCCTTGTTGTTTAGCTTTCTAA 798
                ******************  ******* **********************
```

Figure S3. Projection of exon boundaries on protein sequence of GγC subunits from selected eukaryotic species; conserved DPLL motif highlighted in green; hyphen and amino acids highlighted in yellow display positions of exon boundaries.

Plants

Arabidopsis thaliana AtGGA1 (AGG1)
MREETVVYEQEESVSHGGGKHRILAELARVEQEVAFLE-KELKEVENTDIVSTVCEELLSVIEKGPDPLLPLTNGPLNLGWDRWFEGPNGGEGCRCLIL

Arabidopsis thaliana AtGGA2 (AGG2)
MEAGSSNSSGQLSGRVVDTRGKHRIQAELKRLEQEARFLE-EELEQLEKMDNASASCKEFLDSVDSKPDPLLPETTGPVNATWDQWFEGPKEAKRCGCSIL

Arabidopsis thaliana AtGGC (AGG3)
MSAPSGGGEGGGKESAAGGVSSSSLAPSSLPPPRPKSPPEYPDLYGKRREAARVQMLEREIGFLE-GEIKFIEGVQPASRCIKEVSDFVVANSDPLIPAQRKSRRSFRFWKWLCGPCLSLVSFCCCCQSKCSCHLRKPKCCNCTSCSCIGSKCCDGSCCSNICCCPRLSCPSCSCFRGWCSCPDMSCCIPSCFRSCSCTRPSCLNKKKSSCCSCNCKIRWSSCFSCPKVRLCSCCFCNCKNLCSNPCCLAF

Oryza sativa OsGGA (RGG1)
MQAGGGGDAGDTRGRHRIQAELKKLEQEARFLE-EELEELDKTDKVSAALQELMVTAESKADPLLPVTTGPACQSWDRWFEGPQDLRRCKCWFL

Oryza sativa OsGGB (RGG2)
MRGEANGEEEQQPPRRNHLRDDAEEEEEVERRAARPVSGQQQQQQRRRPTDVGGGAAMRSVGYVGKHRLSAAIARLDQELQSLQ-DELNELETMEPASAACQGVITSTEGKSDPLLPVTIGPENASWERWFQRVRSSRSNKWWASKGSDFS

Oryza sativa OsGGC1 (GS3)
MAMAAAPRPKSPPAPPDPCGRHRLQLAVDALHREIGFLE-GEINSIEGIHAASRCCREVDEFIGRTPDPFITISSEKRSHDHSHHFLKKFRCLCRASACCLSYLSWICCCSSAAGGCSSSSSSSFNLKRPSCCCNCNCNCCSSSSSSCGAALTKSPCRCRRRSCCCRRCCCGGVGVRACASCSCSPPCACCAPPCAGCSCRCTCPCPCPGGCSCACPACRCCCGVPRCCPPCL

Oryza sativa OsGGC2
MGEAPRPKSPPRYPDLCGRRRLQLEMQILNREVGFLE-QELQGLERIQPVSRCCKDVNEFVGAKSDPLIPINKRKHRSCSLYRWIRSKLCNCLLCLCCWCRCLPKPKKPSCFSCSCCSCCDTSCCRPSCGCLKAPSSCCCKSNCSCCSSDCCTCSLPSCGCTGCGHCRPLCGGGGGCCPPSDCCSSCKCSCSSCTRCCSSCAGGCKPSCSGCGTGCSSCGGGCCPKCSSCAAPCVGCLALLRRWLSCRSSCCKGQPSCCKCQSSCCEGEPSCCCCCGGGKGSSACCCGRPCCLGGATPAPSCPECSCGCSCSCPRCKDGCSRPSCGNPCCAGGCLC

Oryza sativa OsGGC3 (DEP1)
MGEEAVVMEAPRPKSPPRYPDLCGRRRMQLEVQILSREITFLK-DELHFLEGAQPVSRSGCIKEINEFVGTKHDPLIPTKRRRHRSCRLFRWIGSKLCICISCLCYCCKCSPKCKRPRCLNCSCSSCCDEPCCKPNCSACCAGSCCSPDCCSCCKPNCSCCKPNCSCCKTPSCCKPNCSCSCPSCSSCCDTSCCKPSCTCFNIFSCFKSLYSCFKIPSCFKSQCNCSSPNCCTCTLPSCSCKGCACPSCGCNGCGCPSCGCNGCGCPSCGCNGCGLPSCGCNGCGSCSCAQCKPDCGSCSTNCCSCKPSCNGCCGEQCCRCADCFSCSCPRCSSCFNIFKCSCAGCCSSLCKCPCTTQCFSCQSSCCKRQPSCCKCQSSCCEGQPSCCEGHCCSLPKPSCPECSCGCVWSCKNCTEGCRCPRCRNPCCLSGCLC

Brachypodium distachyon ADDN01000059
MQVPGGGGGGGAGREAGDTRGRHRIQAELKKLEQEARFLK-EELQELEKTDIISAALQEFLVTIEGKADPLLPVTTGVAYQSWDRWFEGPEDLRRCKCWCL

Brachypodium distachyon ADDN01000765
MRGEANGEGRGEEEQQQQQVQEGEEADGAARPSSGQQQPAAAAAATTRGVGYVGKHRLSAAIARLDQELQSLQ-DELNELETMEPASAACQEVITSTQGKPDPLLPITSSPENSSWDRWFQRVPSSRSSKWWTSKGSNFS

Brachypodium distachyon ADDN01000372
MVAPRPKSPPASPDPCGRHHLQLAVDTLHREIGFLE-GEISSVEGVHAASKCCKEVDEFVGKNADPFITISSKKANTDQSRHLPKKFRARTCLSYLSWMCCCGGCPSVQLQGPTSCCSCGALGGLCGCCSTGECCRCRVGCGGCGCCCCCRGSPCRSRTPSPRCSCGCTCSCPSCCSSSCACPAPSCCRAPRCCYLCS

Jatropha curcas BABX01002540
MDSETASSVDEQVGAAGASVGAGADTRGRHRILAEVKRVEQEIKCLE-EELEELEKTDNVSTICEELLRNVQISPDPLLSETIGPVNPLWDRWFEGPQESQGCRCWIL

Carica papaya ABIM01007700
MAVPPGGGSGVPSLPPPCPKSPPEYPDLYGKRRGMAKVQLLEREIGFLE-EEVKSVQALQPASKCCKEVTDFVVARPDPLIPTKHKKHRRSCRFWKWLCGLPCFSLSWICCCCHSQCSCQVKCPQCPRCSDCLKCPQCPRCFQCPKCPKCPKCPRCCDCNCDCGCHCNICSCKSCFSCPRVKCGWPQCCCSCACPSLPSCCCRKISCSWKCCSFRLPSCPDCSSCCRWGCSCPKCPKPKVRLCCSCKQLCCNPCCLFF

Fungi

Laccaria bicolour ABFE01000145
MNARPHK-QSMSELKLRRLTEHNQRLREDLARPRVRVSEASTSLIRYCKTTKDHL-VPSVWGPVGKNEDPYAPPAQGCNCVIM

Aspergillus clavatus DS027059
MAPYELRSGGDVKNKKQSVADLKYRRLTELNARLKEDLDRPRVKVSEASMSLINYCNNTRDFMVPSVWGQ-VDKREDPYAPQQQGGCCTVM

Stramenopiles *Phytophthora infestans* AATU01005457
MSDAASKKLSEEIARLEIDLKTLEASCTTSEAAKKIAEYCQSTADPFLGENDSGPNPWQQSGQGGGGCSIL

Amoebozoa *Dictyostelium discoideum* AAFI02000012
MSESQLKKVLKENETLKAQLEKSTTILKVSEACESLQDYCTKTSDPFIPGWSGENEWTKPLKGNGCSVL

Ichthyosporea *Capsaspora owczarzaki* (Holozoa) ACFS01000456
MVDQKTERMKKTVDALSAELKFNRIKVSEAAKLLKDYCKDTKDPLIPSIWGRLDDKENPYAKDKGKGCLVL

Animals

Ixodes scapularis ABJB010297719+ ABJB010087644
MSTLQQQRKVVEQLRREAALKRMEVSTAIEDLK-KYIREHENDYLLVGFHSQKANPFREKSSCILL

Caenorhabditis elegans Z67883 (gpc-1)
MENIKASTEQLCAEANIQRKKVSEVSKELLDFCEKNKTNDMLVSGPTDQHNPFQEKKSCSVL

Saccoglossus kowalevskii ACQM01040230
MSSAKAAAEQNVAALKYQLNIERIPASKSIKELVQYIQDNEPTDPFLHPVDKKENPWADRGKCSIL

Branchiostoma floridae ABEP02001366
MSSWGKSQDNAVLIMKKQVEQLRREALMERVKVSQAAADLRNYTQQNAQHDGLLVGIPSSQNPFKESTKCNIL

Danio rerio CABZ01009074
MSTTNNIAQARKLVEQLRIEAGIERIK-VSKAAADLMSYCKQHARSDPLLVGVPTSENPFKDKKPCIIL

Xenopus tropicalis AAMC01111108+AAMC01111109
MSTSNNIAQARKLVEQLRLEAGIERIK-VSKAAADLMNYCEQHAKNDPLLVGVPTSENPFKDKKPCSIL

Homo sapiens GNGT1
MPVINIEDLTEKDKLKMEVDQLKKEVTLERML-VSKCCEEVRDYVEERSGEDPLVKGIPEDKNPFKELKGGCVIS

Homo sapiens GNG12
MSSKTASTNNIAQARRTVQQLRLEASIERIK-VSKASADLMSYCEEHARSDPLLIGIPTSENPFKDKKTCIIL

Table S1. Amino acid content (%) and approximate length of the cysteine-rich domain of type C Gγ subunits

Species / amino acids	A	R	N	D	C	E	Q	G	H	I	L	K	M	F	P	S	T	W	Y	V	Number of aa in C-tail
**Gymnosperms**																					
Cycas rumphii DR061731	2.9	8.7	1.4	4.3	18.8	2.9	1.4	1.4	5.8	5.8	2.9	10.1	0	8.7	2.9	12	0	7.2	1.4	1.4	69
Zamia furfuracea CB095456	0	13	0	4.3	20.3	2.9	1.4	0	2.9	7.2	4.3	10.1	1.4	8.7	1.4	12	1.4	5.8	0	2.9	69
Picea sitchensis DR533730	2.6	6.6	3.9	1.3	23.7	1.3	1.3	1.3	2.6	5.3	2.6	14.5	0	9.2	2.6	9.2	1.3	6.6	1.3	2.6	76
Picea glauca DR579171	2.6	6.6	3.9	1.3	23.7	1.3	1.3	1.3	1.3	5.3	2.6	14.5	0	9.2	2.6	9.2	1.3	6.6	2.6	2.6	76
**Dicots**																					
Arabidopsis thaliana AtGGC1 (AGG3)	0.7	4.9	4.9	1.4	32.2	0	0.7	2.8	0.7	2.8	6.3	7.7	0.7	4.2	6.3	18	1.4	2.8	0	1.4	143
Brassica rapa AC189411	1.2	4.3	4.9	0.6	34.1	0.6	0	2.4	0.6	1.2	5.5	8.5	0	5.5	8.5	17	1.8	1.8	0.6	1.2	164
Aquilegia formosa DT735500	0.9	3.8	4.7	0	31.1	0.9	0.9	3.8	1.9	0.9	5.7	11.3	0	5.7	5.7	12	2.8	4.7	0.9	1.9	106
Glycine max CX701891	4.8	6.5	4.8	1.6	30.6	0	3.2	3.2	3.2	1.6	6.5	8.1	1.6	1.6	1.6	16	1.6	1.6	0	1.6	62
Medicago truncatula AC169626	3.3	1.7	5	0	21.7	0	0	5	1.7	5	8.3	11.7	0	6.7	3.3	18	3.3	1.7	3.3	0	60
Glycine max FG994755+BT095007	0	2.2	2.9	0.7	36.8	1.5	0.7	4.4	0	1.5	5.9	7.4	0.7	5.1	11	11	3.7	3.7	0	0.7	136
Medicago truncatula AC202480	0	0.7	5.8	1.4	32.4	0	0.7	2.9	0.7	0.7	6.5	6.5	0.7	5.8	9.4	15	5	3.6	0.7	1.4	139
Populus trichocarpa DT488475	0.8	5	5.8	0.8	34.2	1.7	0.8	1.7	1.7	3.3	7.5	5.8	0	2.5	9.2	10	4.2	4.2	0	0.8	120
Solanum lycopersicum BI210240	0	3.3	5.8	0	34.7	0	0.8	0.8	0	2.5	5	8.3	1.7	3.3	9.1	16	2.5	4.1	1.7	0.8	121
Solanum tuberosum BQ116994	0	3.4	5	0	34.5	0	0.8	0.8	0	2.5	5.9	8.4	1.7	2.5	9.2	16	1.7	4.2	2.5	0.8	119
Centaurea maculosa EH739324	0.8	2.4	3.9	2.4	37.8	0	0	3.1	0	1.6	4.7	7.9	0.8	4.7	10.2	15	1.6	2.4	0.8	0	127
Raphanus raphanistrum FD976826+FD981034	2.2	4.4	4.4	1.1	33.3	1.1	1.1	4.4	1.1	2.2	7.8	5.6	0	3.3	7.8	14	2.2	2.2	0	1.1	90
Gossypium raimondii CO121496+CO121497	0.4	1.8	4.7	3.3	34.2	0.4	1.1	1.8	1.1	2.5	5.5	9.1	0	2.5	4.4	22	2.2	2.2	0.7	0.4	275
Vitis vinifera AM427921	0.5	5.9	6.4	2.7	33.2	0.5	0.9	0.9	0.9	2.7	4.5	4.5	0.9	6.8	6.4	18	0	3.2	0.5	0.5	220
Beta vulgaris FG344262	1.3	8	5.3	1.3	29.3	2.7	6.7	4	2.7	1.3	8	10.7	0	2.7	1.3	11	0	1.3	0	2.7	75
Curcuma longa DY386604	1	8.8	2.9	0	23.5	1	0	3.9	2.9	3.9	2.9	2.9	1	3.9	6.9	17	6.9	3.9	2	4.9	102
Zingiber officinale DY350004	1	4	1	1	26.7	2	0	5.9	2	3	5	5	1	2	5.9	18	5	5	3	4	101
Elaeis guineensis EL690747	1.9	13.2	4.7	0	31.1	1.9	1.9	2.8	0.9	3.8	5.7	5.7	0.9	1.9	5.7	9.4	2.8	4.7	0	0.9	106
Cenchrus ciliaris EB660797+EB671123	9	7.4	0	0.8	30.3	0	0.8	10.7	0	0.8	5.7	4.1	0.8	2.5	7.4	14	1.6	1.6	1.6	0.8	122
**Monocots**																					
Sorghum bicolor XM_002465107	9.5	8.6	0	0.9	29.3	0	0.9	12.1	0	0.9	7.8	4.3	0	1.7	8.6	8.6	0.9	1.7	2.6	1.7	116
Zea mays NM_001151000	9.6	7	0	0.9	27.8	0	0.9	6.1	0	0.9	7.8	4.3	0.9	2.6	11.3	11	1.7	2.6	2.6	1.7	115
Oryza sativa CT835094 OsGGC1(GS3)	8.8	8.8	2.7	0	32	0	0	6.8	0	0.7	4.8	2.7	0	2	8.8	17	1.4	0.7	0.7	2	147
Sorghum bicolor XM_002444424	5.3	4.8	1.9	0.5	31.6	1	1.4	10.5	1	0.5	3.8	1.9	0.5	1.4	7.7	19	5.3	1	0	1.4	209
Saccharum officinarum CA230676+CA230756	6.6	5.6	1.5	1	31.3	1	1.5	10.1	1	0.5	4	2.5	0.5	1.5	9.1	17	4	0.5	0	0.5	198
Zea mays EU976637	3.3	5.7	1.4	0.9	30.2	1.9	1.4	10.8	0.9	0.9	4.7	2.8	0.5	1.9	9.4	18	4.2	0.9	0	0.5	212
Triticum aestivum CJ638838+CJ666924	2.4	3.8	2.4	1.9	33.7	2.9	2.9	10.1	0	1.4	3.4	5.8	0	2.9	7.2	15	1.4	1.9	0	0.5	208
Sorghum bicolor XM_002460230	2.3	2.6	5.5	2.3	33.8	0.6	1.9	4.5	0.3	0.6	3.2	7.1	0	2.9	8.8	18	1.9	2.3	0	1	308
Zea mays NM_001158725	1.6	3.4	4.4	2.8	32.8	0.9	1.9	5	0.3	0.9	2.8	7.2	0.3	3.8	8.4	18	1.6	1.6	0.6	1.3	320
Phyllostachys edulis FP100709	0.6	3.1	5.1	1.7	33.7	2.9	2.6	4	0	1.4	1.7	6.9	0	3.4	8	21	2.6	0.9	0.6	0.3	350
Oryza sativa NM_001069822 OsGGC3(DEP1)	1.8	2.7	4.7	1.5	34.7	1.8	2.4	7.1	0.3	1.8	3	6.2	0	2.7	8.3	17	2.7	0.6	0.6	0.3	337
